# Perspectives of adults with Klinefelter syndrome, unaffected adolescent males, and parents of affected children toward diagnosis disclosure: a Thai experience

**DOI:** 10.1007/s12687-019-00435-6

**Published:** 2019-09-04

**Authors:** Sukrit Suwannachat, Duangrurdee Wattanasirichaigoon, Jiraporn Arunakul, Vilawan Chirdkiatgumchai, Thipwimol Tim-Aroon

**Affiliations:** 1 Division of Medical Genetics, Department of Pediatrics, Faculty of Medicine Ramathibodi Hospital, Mahidol University, 270 Rama 6 Road, Bangkok, 10400 Thailand; 2 Division of Child and Adolescent Health, Department of Pediatrics, Faculty of Medicine Ramathibodi Hospital, Mahidol University, Bangkok, 10400 Thailand; 3 Division of Child Development, Department of Pediatrics, Faculty of Medicine Ramathibodi Hospital, Mahidol University, Bangkok, 10400 Thailand

**Keywords:** Klinefelter syndrome, 47,XXY, Genetic counseling, Children, Disclosure of diagnosis, Asian perspective

## Abstract

Little is known about disclosure of diagnosis to males with Klinefelter syndrome (KS) diagnosed before birth or during childhood, especially in Asian context. Insufficient preparation for disclosure communication could lead to the child’s depression, anxiety, or disrupted self-esteem. This is an interventional and qualitative interview study with a brief self-reported survey. The study consists of three phases. In phase I, adults with KS were asked about their experiences and viewpoints toward KS disclosure and diagnosis through individual interviews. In phase II, unaffected adolescent males were given information about KS and then asked to give hypothetical answers to a questionnaire followed by a group interview, as if they had KS. In phase III, parents of children with KS were asked to complete a questionnaire followed by a group interview which included a discussion about their disclosure plans and their concerns. The parents were provided information obtained from adults with KS and unaffected adolescent males, and were then interviewed again as a group. We found that the most hurtful issues were discovering their infertility from KS and late disclosure. From the perspective of unaffected adolescent males, early disclosure in early or mid-teens in a neutral, supportive, and relaxed manner, along with information on benefits of hormonal treatment, the assurance of biological sex as “male,” and avoidance of some sensitive words are strongly recommended, as a way to promote self-confidence and positive coping with the diagnosis. After intervention, the parents reported more confidence and less anxiety about disclosing the diagnosis to their children, and indicated that they would disclose KS at an earlier age. For unaffected adolescent males, though it is a hypothetical scenario and their reaction may differ from affected adolescents, unaffected adolescent males’ viewpoints on how they might react are more or less representative of cultural changes for the new generation, especially in the Asian context. In addition, their viewpoints influenced parents’ decision and genetic counseling plans for the diagnosis disclosure of KS to their children. We propose a preliminary guideline for KS diagnosis disclosure.

**Electronic supplementary material:**

The online version of this article (10.1007/s12687-019-00435-6) contains supplementary material, which is available to authorized users.

## Introduction

Klinefelter syndrome (KS) or 47,XXY syndrome is one of the most common sex chromosomal disorders, with a prevalence of 1:500–1:1000 male births (Nielsen and Wohlert 1990; Visootsak et al. 2001; Visootsak and Graham Jr 2006). Generally, males with KS are born with normal external genitalia and have normal psychomotor development during the infantile period. About 10% of children with KS experience learning disabilities and/or language problems during childhood (Bruining et al. 2009). The extra X chromosome leads to testicular fibrosis and subsequent small testes, underproduction of testosterone, and reduced fertility (Visootsak et al. 2001; Visootsak and Graham Jr 2006). In adolescence, children with KS experience delayed and incomplete puberty; breast enlargement (in one-third of individuals with KS); reduced facial, axilla, pubic, and body hair; reduced muscle mass and strength; and decreased libido (Visootsak et al. 2001; Van Batavia and Kolon 2016). Androgen replacement therapy is prescribed for some individuals with KS usually starting at around age 12 (Visootsak and Graham Jr 2006).

The majority (64%–75%) of individuals with KS remain undiagnosed (Abramsky and Chapple 1997; Bojesen et al. 2003; Nieschlag 2013). Around one-fourth of those diagnosed postnatally are diagnosed during childhood or adolescence because of learning and behavioral problems and/or hypogonadism, with the remainder diagnosed in adulthood because of infertility/hypogonadism (Abramsky and Chapple 1997; Visootsak and Graham Jr 2006). The number of children with KS diagnosed prenatally is predicted to rapidly increase following amniocentesis indicated by advanced maternal age and/or positive noninvasive prenatal testing (NIPT) in general pregnancy. This will increase the need for prenatal genetic counseling on KS and a guideline for disclosure of a KS diagnosis to this group of children. The disclosure of KS diagnosis to these children can be challenging for their parents and physicians (Girardin and Van Vliet 2011; Dennis et al. 2015). Few studies have focused on how to disclose a KS diagnosis to this group, and there is no consensus on guidelines for diagnosis disclosure (Stochholm et al. 2006; Dennis et al. 2015; Tremblay et al. 2016; Turriff et al. 2017). It has been suggested that healthcare professionals (HCPs) partner with parents to disclose KS diagnosis to their child (Tremblay et al. 2016). When approaching adolescence, children with KS may undergo multiple medical appointments and genital examinations for hormonal replacement therapy. Leaving children with KS uninformed may lead to fear, distrust, and disrupted family relationships (Sullivan and McConkie-Rosell 2010).

There are limited studies from Asian countries on how cultural differences may influence parental decision-making and genetic counseling. In addition, the level of maturity of children in Thailand and many Asian countries may differ from most Western countries due to cultural and societal differences. In Thailand, the number of affected boys diagnosed as a result of prenatal testing as opposed to postnatal testing based on clinical problems is not known. In our experience at Ramathibodi Hospital during 2000–2017, only 16 cases were diagnosed at prenatal onset and only one case at postnatal setting as an incidental finding from an investigation for congenital anomaly (Pierre-Robin sequence, subsequently confirmed to have Stickler syndrome). There are 17 pediatricians and 7 adult physicians who currently practice clinical genetics in Thailand. These pediatricians and physicians completed formal training in clinical genetics from the UK and the USA. There is no professional genetic counselor in the country. Prenatal genetic counseling is usually provided by maternal fetal medicine specialists or obstetricians who performed prenatal test with or without consultation from clinical geneticists (pediatrics or internal medicine physicians). Postnatal diagnoses lead to genetic counseling by pediatricians and/or pediatric geneticists who encounter the cases. There is no regulation for written informed consent for genetic testing, but consent is required for prenatal testing procedures such as amniocentesis and chorionic villi sampling. In Thailand, there is no guideline for pre- and postnatal counseling of KS. Physicians usually provide counseling based on their previous training, individual experience, and knowledge available in existing literature.

In Thailand, various types of antenatal screening and testing are available, including maternal serum screening, amniocentesis, cordocentesis, and chorionic villi sampling for karyotype analysis, QF-PCR, and recently cytogenomic array and (since 2015) noninvasive prenatal test. None of these tests is reimbursable; therefore, only those who can pay out-of-pocket can afford these tests. Most tests are available at major public and private hospitals and at some smaller hospitals through in-house service laboratories or private laboratories. It was not until September 2018 that the Thai Universal Coverage launched the service of maternal serum prenatal screening for advanced maternal age. Termination of pregnancy at no later than 24-week gestation is legally allowed for maternal physical/mental health and fetus with serious conditions. Most, if not all, people in Thailand are Thai and speak Thai language. Over 95% of Thai people are Buddhists. Most Thai couples accept prenatal diagnosis and termination of pregnancy if the pregnancy is complicated with severe maternal or fetal conditions. According to UNESCO’s Asia and Pacific Regional Bureau for Education, 85% of Thais complete lower secondary education and 62% of this group are girls. At the end of lower secondary education, only 50% have minimum proficiency level in reading, which may indirectly reflect low health literacy (https://bangkok.unesco.org).

The objectives of the present study were (1) to explore perspectives of adult males with KS and unaffected adolescent males, then compare them with parental plans for diagnosis disclosure both pre- and post-intervention with the information given from the affected adults and the unaffected adolescent groups; and (2) to develop a preliminary guideline for parents and HCPs in disclosing KS diagnosis to affected children.

## Methods

**Fig. 1:**
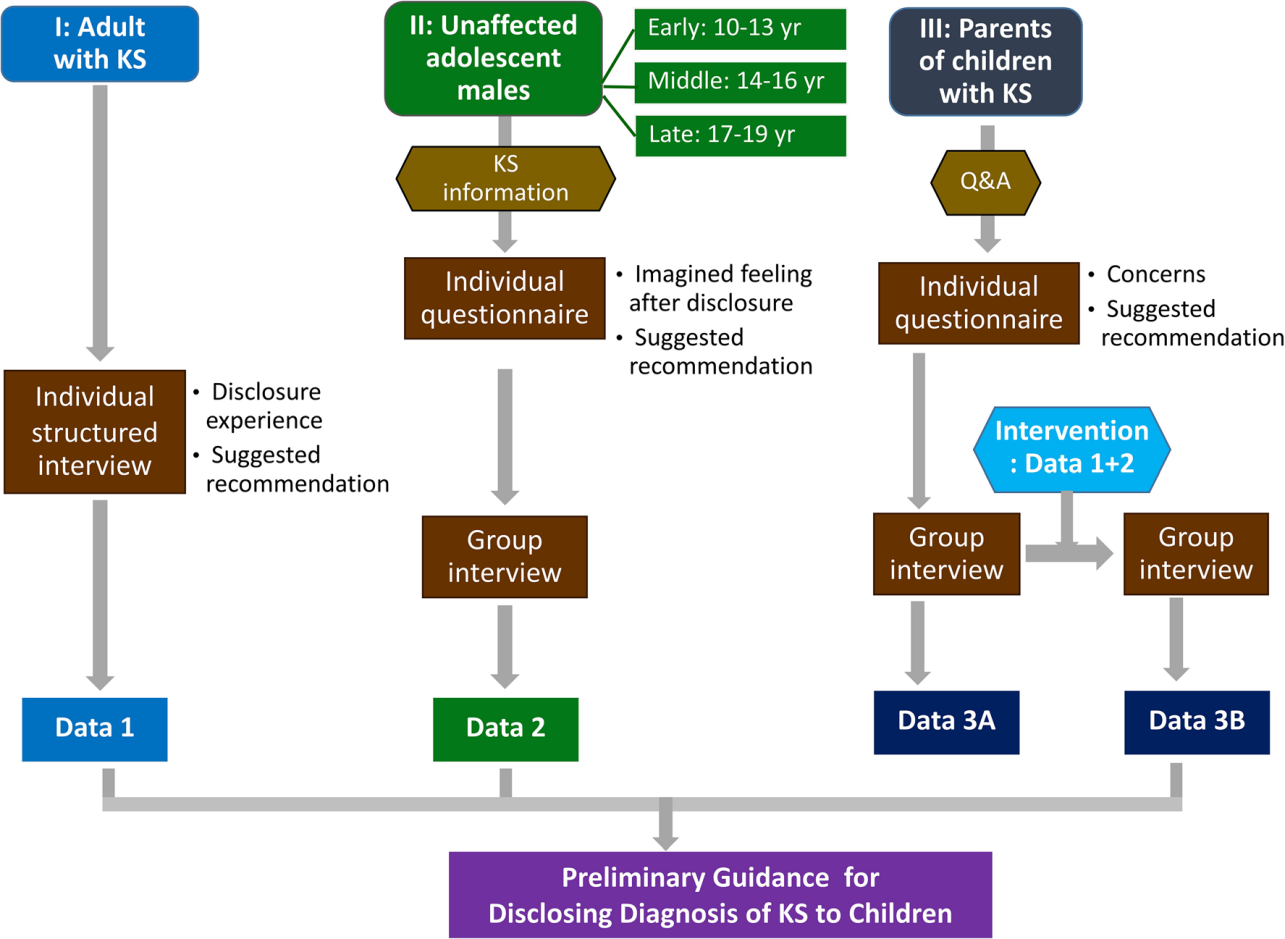
Three study phases. Data collected from adult males with Klinefelter syndrome (KS) in phase I and unaffected adolescent males in phase II were used to inform the parents of children with KS, followed by a group interview with parents. Guidance for KS disclosure was then developed

This is an interventional and qualitative interview study with a brief self-reported survey, consisting of three phases, each with a different participant group. Phase I involved adult males with KS, phase II involved unaffected adolescent males, and phase III involved parents having a child with KS (Fig. 1). This study was conducted between June 2016 and September 2017. Eligible cases were children diagnosed with KS during 2005–2016. To avoid/minimize an inherent problem in translation questions back and forth between Thai and English language, a Thai layperson who is fluent in both languages performed back translation of the information asked to/responded from the study subjects. We then compared the meaning of the sentences used and edited the English presentation as appropriate. We used content analysis by categorizing verbal or behavioral data to classify, summarize, and tabulate the data.

### Phase I: Individual interviews with adult males with KS

There were four adults with KS receiving follow-up from adult genetic/infertility specialists at Ramathibodi Hospital. All were approached and all agreed to an interview. Audio-recording of interview conversations was performed when permitted by participants; otherwise, note-taking by the interviewer took place. Then the data was typed, collected, and analyzed in both quantitative and qualitative formats.

The interview comprised two sections. Part 1 covered individual information and experience of KS diagnosis disclosure, their reactions and feelings when first disclosed, the impact of knowing the diagnosis on their lives, and how they overcome the problems, confidentiality issues, and attitudes toward their diagnosis. Part 2 explored individual recommendations on how the diagnosis should be disclosed. Each interview lasted 30–45 min. The data from each individual with KS was obtained in qualitative format. The questions constructed were based on the author’s experience (DW) who had encountered questions/reactions from the parents regarding diagnosis disclosure of KS. Among these, the most frequently asked questions prenatally are whether or not the “extra female chromosome” would affect the child’s behavior. Some parents feared that the affected child would express female-like behavior; therefore, during the preparation of the structured interview questions, we considered this point in the discussion among all the authors including child development specialist (WC) and adolescent specialist (JA). Of note, in Thai lay language, the “X” chromosome is not uncommonly referred to as “female chromosome,” and some of the parents were given prenatal counseling that the fetus had “extra female chromosome.” Information from these interviews was used in preparing interview questions for phases II and III of the study.

### Phase II: Questionnaire and group interviews with unaffected adolescent males

Because we would like to disclose the diagnosis to affected children and that infertility is a major concern among adults with KS, we explored viewpoints of unaffected adolescent males, not adults. Adolescent males differ from general adult population in several aspects. First, they are more representative of cultural changes experienced by the new generation, which places less significance on having children as a defining characteristic of having a family. Second, hormonal replacement therapy is recommended to start at around puberty, which could create confusion and unnecessary anxiety or fear among adolescents with KS about to receive treatment. Therefore, we sought to obtain the viewpoints of unaffected males who are close to the age range of the affected males to be disclosed.

The information package was distributed to adolescent males aged 10–19 who attended a summer soccer camp in Bangkok, where there was a mix of students from various schools. These teenagers were asked to share the information with their parents. After 2 weeks, information sheets and parental consent forms were distributed to adolescents who expressed interest in participating in the study. We recruited three groups of seven adolescent males: in early (10–13 years), middle (14–16 years), and late (17–19 years) adolescence. Two weeks later, adolescents whose parents provided written informed consent were invited to Ramathibodi Hospital to complete the questionnaire and participate in a group interview. Each group met separately with the research team.

#### Questionnaire

During the group meeting, information about KS was delivered by the research team via a PowerPoint presentation and a one-page document about KS. This included chromosome findings in KS; clinical manifestations at birth and in later life; the impact of KS on physical and intellectual development, learning, and reproductive functions; and treatment regime. This was followed by a 20- to 30-min question and answer session.

Next, participants completed a one-page questionnaire comprising six items, including (1) their age and prior knowledge of KS; (2) imagining that they had KS, how they would like the diagnosis to be disclosed to them, including when, what, and how; (3) words to be used and avoided; (4) their possible feelings if KS was to be disclosed; (5) their willingness to share this information with others; and (6) how infertility due to KS would affect their future plans for dating and marriage. The questionnaire took 10–15 min to complete. Of note, we did not ask the adolescents or their parents about their medical history.

#### Group interviews

The 80- to 90-min group interviews were led by adolescent and genetic specialists (JA and DW, respectively). Based on the hypothetical situation that participants were affected with KS, interview questions included what information they would want to know, when, and at what age; advantages and disadvantages of early versus late disclosure; words to avoid; their attitudes toward having “an extra female chromosome”; whether knowing about the physical manifestations of KS (less hair, possible enlarged breasts, small-sized penis) would affect their self-confidence; how they would cope with the diagnosis; what type of support would be most important to help them overcome challenges; and confidentiality issues. The interview also included a discussion about how they would react to a friend affected with KS, if they were an unaffected male. The interviews were audio-recorded then transcribed into text and analyzed. Common keywords with similar meaning from individual participants were selected and counted. Key sentences were recorded.

### Phase III: Parent questionnaire and group interview

Over 12 years of our clinical service in Thailand, there are now17 families having a child with KS. Two families have lost contact. Among the 15 families invited to participate, 10 families were able to join the entire process of the study (questionnaire and group interview). We invited both parents and initially hoped to analyze the answers into two subgroups, father vs mother, to see the difference (if any) between paternal and maternal viewpoints. However, some couples wanted to give one answer for both parents, and, in some cases, only one parent from the family was available to take part in the study. Therefore, we decided to count each individual response as one parental participant.

#### Questionnaire

First, 15–20 min was allocated to answering any questions about KS in a group format. Each parent was then asked to complete a one-page questionnaire comprising six items, including their relationship to the child; the age of the child; whether or not the child was aware of his KS diagnosis; parental plans to disclose the diagnosis to the child, at what age/school year, and by whom; rating of concerns about the disclosure process (the child’s feelings, suitable age for disclosure, appropriate words to use, infertility issues, the child’s ability to understand what was being explained to them, confidentiality, gender identification and possible confusion, physical health, and others); and predicted reactions of their child following the disclosure (relief, anxiety, curiosity, neutral, panic, anger, sad, etc.). The questionnaire took 10–15 min to complete.

#### Parents’ group interview

The interview lasted 80–90 min and was based on 15 leading questions covering understanding about KS; answers to the child with KS when asked about the reason for routine hospital visits; if they had not disclosed the diagnosis to their child, what was the major reason(s); impact of early versus late disclosure on the child/family; what information they would give; the easiest and most difficult topics to explain to the child; how they would handle this difficult situation; the most important factor to help their child cope with the diagnosis; the most serious reaction from the child that parents would consider a “red flag” or cry for help; and the kind of help parents need from physicians in disclosing KS to their child.

At the end of the interview, information obtained from the adult males with KS and unaffected adolescent males was provided to the parents by a 15-min PowerPoint presentation followed by open discussion and a question and answer session in a group format for another 15–30 min. Parents were then asked whether this information impacted their attitudes, concerns, anxiety, and plans related to the disclosure of a KS diagnosis to their child. The interviews were audio-recorded then transcribed into text and later tabulated as needed before analysis.

### Ethics

This study was conducted after approval from the Ramathibodi Hospital Institutional Review Board (protocol ID 08-59-01). Parental signed consent was required for teens aged < 18 years before participation in this study. Adult males with KS provided written consent for a face-to-face interview or verbal consent for a telephone interview. Participating parents of affected children also gave written consent.

## Results

### Experiences and individual recommendations of adult males with KS

The four participating males with KS were aged 39, 42, 43, and 23 years. The first three participants were diagnosed following infertility investigations, and the last at age 15 years because of delayed secondary sex characteristics and a small penis. All four participants had received genetic counseling from infertility specialists and/or geneticists. Participants gave permission for telephone interviews without audio-recording. Two individuals reported they would have preferred KS to be disclosed earlier so that they could prepare themselves, one felt indifferent, and one was unsure if he wanted to be informed earlier. When first finding out about their diagnosis, three individuals felt sad, shocked, grieved, very upset, and disappointed in themselves, along with feelings of being imperfect.

For the three older participants, knowing that they were infertile due to KS was the most upsetting, whereas late disclosure was the most hurtful for the youngest participant (Table 1). One individual said he would not have married if he had been aware of his infertility. Another said he might have felt confused about his gender and sexuality if the diagnosis was disclosed to him as a child. Two participants said that, as teenagers, they felt worried for not knowing what was wrong with their bodies (slender physique, decreased body hair and muscle mass, small-sized penis), and that they would have preferred to be diagnosed and informed earlier.

**Table 1:** Concerns and impacts of Klinefelter syndrome (KS) diagnosis and disclosure: viewpoints of parents with affected children, adults with KS, and unaffected adolescent males

Concerns/impact	Parents of children with KS (*n* = 14)	Adults with KS (*n* = 4)	Unaffected adolescent males (*n* = 21)
1. Most concerning	Child’s emotional response and self-esteem (9)	Affect self-confidence (4) Confused about true gender identity (1)	Affect self-esteem (15)
2. Most difficult/hurtful issue to discuss with the child	Infertility (6); Effect of KS on physical appearance (4)	Infertility (3) Late diagnosis/disclosure (1)	Infertility (15) Hearing the words “extra female chromosome” (16)
3. Easiest issue to discuss with the child	Infertility (4) Effect of KS on physical appearance (1)	NA	NA
4. Feeling/reaction of individuals with KS following disclosure	Negative: worried (4) Positive: accepting (4) Unsure (3)	Negative: depressed (1), worried (3)	Negative: shocked, worried (17), sad (9) Positive: relieved (3), neutral (2), curious (9)
5. What can help individuals to overcome this challenging time	Positive/close parent-child relationship (6) Nonfatal nature of the condition Remind the child that he has been able to lead a normal life and will continue to do so	Knowing that KS is a common condition and does not cause serious health problems (1) Support from wife and hope in ART (1)	Telling oneself that the condition is treatable (7) Comforting themselves (9): “I am a bit different,” “There are people out there who can live with KS, so I can live with it too” Consult a psychiatrist (1)
6. Reactions of the child when informed of having “extra female chromosome”	NA	Confusion on gender identity and fear of gender deviation (4)	Disappointed (8) Should not be mentioned (7) Confusion of gender identity and fear of gender deviation (2)
7. How infertility interfered with future plans for dating and marriage	NA	Decline to pursue marriage (1) Disclose to partner before proposing	Dating: opted (12), declined (8) Marriage: opted (11), declined (10) Disclose to partner before proposing (11)^a^
8. Confidentiality	Suggest keeping the diagnosis confidential	Up to the child (3) Keep it confidential (1)	Keep it confidential (14) Share with others (7)^b^
9. Words to avoid	NA	Syndrome, disease (1) Extra female chromosome (2)	Infertile Extra female chromosome Abnormal, incurable
10. Words to use	NA	Treatable, nonfatal Extra X chromosome	A condition, NOT disease Difficulty having children Extra chromosome, or extra X chromosome Treatable, nonfatal, not serious condition

^a^All teens who opted marriage; ^b^All late teens

NA, data not available or not in the interview questions

*ART* assisted reproductive technology

I am not sure if I want to be told earlier. I might have been confused if I were told earlier as a child.—a 43-year-old KS adult

I might not have gotten married if I were aware of this condition and its related infertility problem. What is the point of marriage if I cannot have my own biological children?—a 42-year-old KS adult

I felt disappointed in myself. For my whole life before knowing the diagnosis, I had thought that I was physically perfect. When first informed of having KS, I felt that I was disabled.—a 23-year-old KS adult

I felt that something might be wrong with my body. I had a slender body like a girl. I did not have muscle, mustache or body hair. … I was depressed after being informed by my OB doctors about the diagnosis. I browsed the internet and found a website containing information about KS. After finding out that KS is a common condition with prevalence of 1 in 800 males, it made me feel better. However, the information available on the website was quite difficult for me to understand even though I was already an adult, so it would be much more difficult for a KS child to understand. We need to have a good resource of age-appropriate information for early disclosure.—a 42-year-old KS adult

Participants’ recommendations regarding KS disclosure were (1) disclosure by both parents before puberty and/or the beginning of hormonal treatment, in a calm and relaxed manner; (2) no need to mention “Klinefelter” or “47,XXY” and the mechanisms underlying the condition because it is an English term which is difficult to understand for boys. In addition, focusing on the possible effects of KS and how to help them would be more helpful. The name of the condition or its mechanism can wait to be explained when the boys are more mature; (3) discuss the effects of KS on physical appearance, breasts, and penis; (4) discuss hormonal therapy and its outcomes; (5) infertility may or may not be mentioned initially, but should be disclosed before the individual starts planning to have their own family; (6) avoid the words “extra female chromosome” (suggested by two participants); and (7) let the child choose whether or not to keep the diagnosis a secret. The youngest participant emphasized that early diagnosis and disclosure would yield better outcomes, and that it was important to reassure the child about the nonfatal nature of the condition.

### Questionnaire results from unaffected adolescent males

In total, 21 unaffected adolescent males participated in this study. None had ever heard about KS; therefore, we presumed that none had known diagnosis of KS. Imagining that they had KS, they wanted the diagnosis to be disclosed by one or both parents together (76%, 16/21), and disclosure during age 9–14 years (90%, 19/21). In addition, 24% (5/21) expressed neutral/positive reactions (relief), 76% (16/21) expressed shock at the news, and 43% (9/21) reported curiosity about KS. Among 43% (9/21) who expressed the feeling of sadness, there were 6, 1, and 2 boys from the early teen, mid-teen, and late-teen groups, respectively (Online resource 1, ST1). Besides the parents, 62% (13/21) of the teens would keep the KS diagnosis secret, whereas the remainder, especially middle and late teens, would share the diagnosis with other relatives and close friends. Over half (57%, 12/21) said they would participate in dating and get married as usual. The older teens had more positive answers on this issue (Online resource 1: ST1).

### Unaffected adolescent males’ viewpoints on what, when, and how to disclose the KS diagnosis

The group interviews revealed that the most desired knowledge about KS included treatment availability and outcomes (48%, 10/21), effects of KS on their physical appearance/health and its severity (43%, 9/21), and their true biological sex assignment (14%, 3/21). A majority of the teens (76%, 16/21) preferred early disclosure before hormonal therapy started (Online resource 1: ST2). Perceived advantages of early disclosure included gradual adaptation to the diagnosis and better cooperation with treatment. Perceived disadvantages were unnecessary anxiety and being bullied by peers. Late disclosure may lead to disadvantages such as greater difficulty accepting the diagnosis (33%, 7/21) and delayed treatment (19%, 4/21), but has the advantage of possibly bypassing years of sadness and being bullied. Half (10/21) of the teens preferred total disclosure, meaning all aspects of the condition should be disclosed to the child at the same time so that the child could then plan their life. The remainder (52%, 11/21) preferred partial disclosure because young children may have difficulty understanding a lot of information at once and understanding concepts such as “infertility” and “chromosomes.” A majority of the teens (71%, 15/21) preferred disclosure of the KS diagnosis by parents in a neutral and relaxed manner (9/21), followed by cheering them up and providing comfort/consolation (9/21). Some adolescents (4/21), especially younger teens, would like their parents to express empathy and accept their feelings of anxiety. Words to be avoided were “infertile,” “abnormal,” “dangerous,” “incurable,” “female chromosome,” and “disease.” Being told that they had “an extra female chromosome” led to negative feelings of disappointment, anxiety, shock, sadness, and discouragement. Some teens suggested using “extra chromosome” instead of “female chromosome.” Only one asked why and where the extra chromosome came from.

To think that I have an extra female chromosome, it gives the feeling of having some bizarre stuff in my body.—an adolescent male

It automatically raises the question of being gay.—another adolescent male

By imagining that they had KS, 57% (12/21) of teens said they would be afraid of being bullied, discrimination by peers, and disruption of normal daily life. In addition, 71% (15/21), especially the late teens, reported reduced self-confidence (Online resource 1: ST3). None of the teens reported concerns about the effect of KS on school performance. Almost all teens in the mid- and late-adolescence expressed interest in future dating and marriage, whereas early teens showed no interest in this issue. Those who were interested in future marriage said they would inform their partner before proposing. The majority of teens (71%, 15/21) appeared to have coped with the KS diagnosis to some extent. The early teen group preferred to keep the KS diagnosis confidential, whereas the middle and late teen groups indicated they would share this information with their close friends and girlfriends. On the assumption that they were unaffected but a friend had KS, participating teens indicated they would react to their friend with normality and empathy.

I am afraid that my girlfriend/spouse would not accept my KS diagnosis and she might worry that we would not be able to have our own biological children.—a middle teen male

I would tell myself that KS is not a deadly condition, that it is treatable, and that treatment has a good outcome.—a teen male

There are people out there with KS who can live with it, so I can live with it, too.—a teen male

It is just that I have something unique.—another teen male

### Results of the parent questionnaire and group interview

Ten out of 15 (67%) families agreed to participate in the study. Although both parents were invited and we were expecting participation from both the father and the mother, ten each, only six fathers and eight mothers, a total of 14 parents, were enrolled in this study. Participating parents were aged 39–53 years. None of the parents had disclosed the diagnosis. The age of their children with KS ranged from 8 months to 10 years, with the majority (7/10) aged between 7 years 9 months and 10 years. At the time of this study, none of the children had received hormonal therapy, although one child was about to start. All children had karyotype 47,XXY from amniocentesis indicated by advanced maternal age.

Parental understanding about problems related to KS was mostly correct, such as tall stature; enlarged breasts if left untreated; possible low IQ and learning problems; delayed speech; personality characteristics including being shy, quiet and sensitive; decreased physical strength; small-sized penis; normal libido (in most cases); reduced fertility; and benefits of hormonal replacement therapy. One-third (4/14) of the parents perceived that there was an increased risk of gender deviance (behavior that violates the norms for gender-appropriate behavior). Planned disclosure of the KS diagnosis was by both parents together (9/14), mother only (3/14), and parents in the presence of physicians (2/14). Eight parents planned to disclose KS during the early teens or before hormonal therapy, five during middle teens, one in adulthood, and one when asked. Two parents reported that their children might know or be suspicious about their KS diagnosis without being disclosed. After disclosure of the diagnosis, parents expected feelings and reactions including curiosity and shock/confusion (11/14), anxiety and worry (6/14), and sadness (3/14) (Online Resource 1: ST3).

Sources of information for parents were treating physicians (86%, 12/14), the Internet (57%, 8/14), books (14%, 2/14), and talking to other parents (7%, 1/14). The most common parental concerns about KS disclosure were the child’s feelings and reduced self-esteem (9/14), the child’s difficulty in understanding the diagnosis disclosure (7/14), infertility (6/14), gender identification and risk of gender deviation (4/14), the impact of KS on the child’s physical health and appearance (1/14), confidentiality (1/14), suitable age for disclosure (1/14), and words to use and to avoid (1/14).

After hearing the information obtained from adults with KS and unaffected adolescent males, parents who planned to disclose the diagnosis during middle or late teen years (7/14) changed their mind and would disclose earlier, and before the start of hormonal therapy. Parents felt better prepared and more confident (14) and had less anxiety (7) in disclosing the diagnosis to their children. Parental plans and concerns were matched to the attitudes, needs, and recommendations of adults with KS and unaffected adolescent males (Table 1). Of note, only a small number of parents chose the suitable age for disclosure and words to use/avoid in the top three concerns during pre-intervention. However, after the intervention, a significant number of parents reported plans of earlier disclosure (7/14) and an appreciation of words to use/avoid (7/14) (Online Resource 1: ST4).

## Discussion

To our knowledge, this is the first study to assess the viewpoints of unaffected adolescent males about KS disclosure, perspectives that may differ from adults with KS. Though it is a hypothetical scenario, the opinions of unaffected adolescent males are more or less representative of cultural changes for the new generation, which places less significance on having children as a defining characteristic of a family in the Asian context. In addition, it has shown influential effect on parental decision and genetic counseling plans for diagnosis disclosure of KS to their children in the Asian context.

All participants agreed that early disclosure would yield more benefit than harm. Important advantages of early disclosure included better adaptation to the diagnosis, better compliance with treatment, and relief about being diagnosed and escaping fears related to uncertainty. Our findings were similar to those found in Western KS-affected population in that the age of diagnosis/disclosure was negatively correlated with adaptation (Turriff et al. 2011; Turriff et al. 2015; Turriff et al. 2017). Tremblay et al. have suggested that disclosure start as young as 5–7 years in a gradual fashion by the parents with or without the presence of HCPs, using analogies and stories to help the child understand easier, be honest, and careful in the words chosen, including psychological follow-up after the disclosure (Tremblay et al. 2016). Although age of diagnosis/disclosure is inversely correlated with adaptation to the diagnosis disclosure, maturity of the child as an individual and within a different cultural context should be taken into account. There is an agreement of the present study and the previous study from Western countries that KS disclosure should be given “before puberty” assuming that this is the second decade. Participants of the study did not discuss what is currently practiced elsewhere, although they suggested age-appropriate explanations.

Similar to the answers of adults with KS, unaffected adolescent males preferred both parents to disclose the KS diagnosis, followed by disclosure by the mother only. This may be because mothers are perceived to be closer to their child and more sensitive to the child’s reaction and emotions. Although adaptation and coping mechanisms developed during childhood may provide a guidance for adolescents with a KS diagnosis, the effect of such a diagnosis on an individual’s life is dynamic and may change with entry to adolescence (Well 2000). Parental and familial support is essential in helping children to cope with the challenges during this vulnerable time (Driscoll 1986; Well 2000). Follow-up conversations and continued monitoring are necessary. Regardless of age, teens were shocked by the information disclosed to them and curious to know more about KS. Therefore, parental preparedness for an open discussion is essential to help the child by answering questions and addressing any fear or anxiety they might have. Encouraging children with KS to share their feelings and thoughts is crucial in helping them to restore their self-image and confidence.

In this study, common parental concerns included the child’s feelings and self-confidence after disclosure of the KS diagnosis, the child’s ability to understand, infertility, gender identification, and risk of gender deviation. A previous study by Dennis and colleagues showed that the most common parental concerns were discussing infertility and the impact on the child’s self-esteem (Dennis et al. 2015). Although 71% of participating teens reported that imagining they had KS would disrupt their self-confidence, they still displayed positive coping mechanisms (Online resource 1, ST3). Younger teens were at higher risk for feeling sad than older teens; therefore, it is important to remind parents that when disclosing a KS diagnosis to their child at a younger age, close monitoring of sadness and continuing encouragement are necessary.

Interestingly, the younger a child was the more likely it was that he would want to keep the diagnosis confidential. This may be because younger teens were unsure about how to deal with this unexpected information. Older teens were better able to choose who they wanted to share the information with and when. This may reflect differences in the child’s maturity stage depending on their age.

Regarding future plans for dating and marriage, the late teens tended to be more positive than the early teens (7/7 vs. 0/7), probably because of greater maturity (Online Resource 1: ST3). Participants with KS who were married reported deep disappointment in hearing about KS-related infertility. They felt responsible for disappointing their spouse in being unable to have biological children, and felt that it was unfair to their spouse to not be informed of KS and its related infertility before marriage. Similar findings were described in the study of Western population, in that infertility, psychological morbidity, and physical appearances were the most common concerns among adults with KS (Turriff et al. 2017). Among the new generation of Thai couples, there is less emphasis on having children. This is based on anecdotal and research data, including a survey of 1320 Thai university students in 2013 which showed that generation Y population in Thailand prioritize work and material achievement, delay the age of having children, and expect to raise fewer children (Samutachak and Darawuttimaprakorn 2014). Therefore, knowing about reproductive difficulties may not be as hurtful for the new generation as for older generations. In addition, early disclosure may alert them before marriage to seek the benefits of advanced assisted reproductive technologies that are making it possible for some people with KS to father children.

All but one teen said that knowing their KS diagnosis would not affect their gender identity. In their opinion, sexual orientation is a result of multiple factors and would have shown some earlier signs. However, they demanded reassurance of their biological sex as “male.” This might have reflected their subconscious concern on gender identification. Some parents (4/14) reported a misunderstanding that KS increased the risk of gender deviation. Borelli et al. also reported that parents of children with KS expressed concerns about their sons’ gender identity and sexual orientation (Borelli et al. 1984). This is a sensitive issue related to the child’s self-image, and the child may be afraid to ask their parents questions, especially in Thai and Asian context where parents and children generally do not have open discussion about sex and gender. Therefore, we suggest that parents’ understanding should be checked for accuracy, and parents should be advised to deliver this message to the child regardless of whether the child asks. Both the parent and teen groups preferred the term “47,XXY” to “Klinefelter syndrome” because it implied a “condition,” not a “syndrome” or “disease.” In addition, both parent and teen groups felt unfamiliar with English term “Klinefelter.” We notice that the word “extra female chromosome” commonly used by laypeople at least in Thailand is a misleading term, creating lots of confusion and fears to the affected individuals, their parents, and the general population. There is an urgent need to educate HCPs in Thailand to refer to the “X” chromosome as “a sex chromosome” and not accept references to it as “a female chromosome.” In addition, HCPs need to correct the misunderstanding through words used among parents and the general population.

Regarding sources of information, most parents obtained data from treating physicians and the Internet, which is consistent with previous reports (Dennis et al. 2015). In this study, the unaffected adolescent males (20/21) chose to go to the Internet for information without having specific websites of interest. They said they would compare information from different websites to confirm their understanding. In this regard, information about KS in the local language and how to disclose KS using lay terms is essential to help individuals with KS acquire accurate and useful data.

As for the benefit of group interview adding on the survey, the group interview allowed us to countercheck the participants’ understanding of questions and answers, explore deeper for the reasons behind their answers, and discover additional questions, concerns, and potential answers not provided in the questionnaire survey. As a result, we found that the unaffected adolescent participants had a correct understanding of the questions and answers. As for the parent group, the post-intervention interview also functioned as a tool to obtain data on whether or not the intervention affected their individual plans of diagnosis disclosure and why. We also asked for consensus on diagnosis disclosure. Moreover, the parent also exchanges their thought to the group.

The present study has a number of limitations. First, the sample size of adults with KS is small, and the interviews of this group were not audio-recorded so it is possible that some data escaped the note-taking process and was excluded in the analysis. Second, unaffected adolescent males imagined a hypothetical scenario and how they might react, which may differ from those adolescents with KS. In addition, they were not asked to report how “real” this hypothetical scenario felt to them which might be useful in gauging how relevant these responses may be in understanding the experience of teens with KS. Third, participating parents had no previous experience in disclosing KS diagnosis to their children.

### Practice implication

Simple guidance for parents and HCPs for disclosure to a child with KS included disclosure by parents in early or middle teen years in a neutral, supportive, and relaxed manner; gradually providing more information over time, with an initial focus on physical appearance, treatment, and outcomes; being clear to state that the child’s true biological sex is “male” to prevent possible gender identity confusion; and that some sensitive words should be avoided whereas others were preferred (Table 2). HCPs have a potentially significant role as a major source of information for parents and provide back-up counseling as needed. Since counseling is a dynamic process, HCPs can also learn from parents about the challenges that arise during the disclosure process. In addition, it is important for parents and HCPs to deal with affected children as individuals, rather than adhere to a rigid guideline. In addition, while the guidelines suggest keeping the diagnosis “private,” it does not mean “secret,” as there are significant psychological consequences of keeping secrets. Parents are encouraged to support the child to share their diagnosis with others as appropriate.

**Table 2:** Simple guidance for parents and physicians for disclosure of Klinefelter syndrome diagnosis to an affected child

Items	Detail
When	1. Early or middle teens, before hormonal treatment starts
How	2. By both parents (NOT doctors), or could be only mother or father 3. In a neutral, simple, supportive and relaxed manner, using pictures or video aids 4. Gradually provide information over time, or complete disclosure depending on the child’s curiosity and maturity
What	5. Focus on the effects of KS on physical appearance and functions, available treatment and outcome, and preparation for future challenges 6. Reproductive difficulty can be mentioned or kept for follow-up conversations, depending on the child’s curiosity and maturity
	7. Be clear to state the true sex identification as “male”
	8. Words to avoid: infertility, abnormal, serious condition, incurable, having extra “female” chromosome
	9. Words preferred: difficulty in having their own biological children, just a little challenge/difference, not a serious condition, treatable, having an extra chromosome
	10. Suggest that children with KS keep the information private, although it can be shared among close friends and relatives

## Conclusion

According to past experiences of diagnosis disclosure among adults with KS, the most upsetting issues were learning about their infertility and late disclosure. From viewpoints of unaffected adolescent males, early and gradual disclosure along with possible treatment options is strongly recommended to promote self-confidence and positive coping with the diagnosis. Both written and audiovisual information for parents and HCPs using simple language for discussion with the child should be developed and made publicly accessible through online resources in a local language.

## Electronic supplementary material

ESM 1 (DOCX 31 kb)
