# Correction to: Perspectives of adults with Klinefelter syndrome, unaffected adolescent males, and parents of affected children toward diagnosis disclosure: a Thai experience

**DOI:** 10.1007/s12687-019-00445-4

**Published:** 2019-11-19

**Authors:** Sukrit Suwannachat, Duangrurdee Wattanasirichaigoon, Jiraporn Arunakul, Vilawan Chirdkiatgumchai, Thipwimol Tim-Aroon

**Affiliations:** 1 Division of Medical Genetics, Department of Pediatrics, Faculty of Medicine Ramathibodi Hospital, Mahidol University, 270 Rama 6 Road, Bangkok, 10400 Thailand; 2 Division of Child and Adolescent Health, Department of Pediatrics, Faculty of Medicine Ramathibodi Hospital, Mahidol University, Bangkok, 10400 Thailand; 3 Division of Child Development, Department of Pediatrics, Faculty of Medicine Ramathibodi Hospital, Mahidol University, Bangkok, 10400 Thailand

**Correction to: Journal of Community Genetics**

10.1007/s12687-019-00435-6

The article **“Perspectives of adults with Klinefelter syndrome, unaffected adolescent males, and parents of affected children toward diagnosis disclosure: a Thai experience”**, written by Sukrit Suwannachat, Duangrurdee Wattanasirichaigoon, Jiraporn Arunakul, Vilawan Chirdkiatgumchai and Thipwimol Tim-Aroon, was originally published electronically on the publisher’s internet portal (currently SpringerLink) on September 2019 without open access.

With the author(s)’ decision to opt for Open Choice the copyright of the article changed on November 2019 to © The Author(s) 2019 and the article is forthwith distributed under the terms of the Creative Commons Attribution 4.0 International License (http://creativecommons.org/licenses/by/4.0/), which permits use, duplication, adaptation, distribution and reproduction in any medium or format, as long as you give appropriate credit original author(s) and the source, provide a link to the Creative Commons licence and indicate if changes were made.

The original article was corrected.

