# Supplementary material for: Perspectives of adults with Klinefelter syndrome, unaffected adolescent males, and parents of affected children toward diagnosis disclosure: a Thai experience
Source: J Community Genet. 2019 Sep 4;11(2):171–81. doi: 10.1007/s12687-019-00435-6 (PMC7062947; doi:10.1007/s12687-019-00435-6)
Supplement: Supplementary file 1 — (DOCX 31 kb) [file 12687_2019_435_MOESM1_ESM.docx]

**Supplementary Table 1.** Unaffected adolescent males’ answers to survey questions

| Items | Early teens  10–13 yrs  (n = 7) | | | Middle teens  14–16 yrs  (n = 7) | | Late teens  17–19 yrs  (n = 7) | | Total  (n = 21) | |
| --- | --- | --- | --- | --- | --- | --- | --- | --- | --- |
| 1. Existing knowledge about KS |  |  | | |  | |  |  |  |
| No | 7 | 7 | | | 7 | | 21 | |  |
| 2. The most suitable person to disclose  the KS diagnosis |  |  | | |  | |  |  |  |
| Father | 0 | 0 | | | 1 | | 1 | |  |
| Mother | 1 | 2 | | | 3 | | 6 | |  |
| Both parents | 5 | 2 | | | 2 | | 9 | |  |
| Parents and physician | 1 | 3 | | | 1 | | 4 | |  |
| 3. At what age (years) should KS  be disclosed |  | |  |  |  |  |  |  |  |
| 9–10 (4th grade) | 0 | 0 | | | 4 | | 4 | |  |
| 10–13 (5th–7th grade) | 5 | 2 | | | 2 | | 9 | |  |
| 13–16 (8th–10th grade) | 2 | 5 | | | 1 | | 8 | |  |
| >16 | 0 | 0 | | | 0 | | 0 | |  |
| 4. Feelings after knowing that they have  KS (≥1 answers) |  | |  |  |  |  |  |  |  |
| Relieved to know the truth | 1 | 2 | | | 0 | | 3 | |  |
| Neutral | 1 | 1 | | | 0 | | 2 | |  |
| Curious | 2 | 3 | | | 4 | | 9 | |  |
| Shock, worry | 6 | 4 | | | 7 | | 17 | |  |
| Angry | 0 | 0 | | | 0 | | 0 | |  |
| Sad | 6 | 1 | | | 2 | | 9 | |  |
| 5. Keep the diagnosis secret or share |  | |  |  |  |  |  |  |  |
| Keep it secret | 7 | 5 | | | 1 | | 13 | |  |
| Share with some others | 0 | 2^a^ | | | 6^b^ | | 8 | |  |
| 6. Plan on future dating |  |  | | |  | |  |  |  |
| Yes | 3 | 4 | | | 5 | | 12 | |  |
| No | 4 | 2 | | | 2 | | 8 | |  |
| Unsure | 0 | 1 | | | 0 | | 1 | |  |
| 7. Plan on marriage |  |  | | |  | |  |  |  |
| Yes | 3 | 3 | | | 5 | | 11 | |  |
| No | 4 | 2 | | | 2 | | 10 | |  |
| Unsure | 0 | 2 | | | 0 | | 2 | |  |

^a^ Two relatives; ^b^ Two relatives and four friends.

KS: Klinefelter syndrome.

**Supplementary Table 2.** Unaffected adolescent males’ viewpoints on WHEN and HOW to disclose Klinefelter syndrome to affected children

| Open-ended questions | Early teens  (n = 7) | Middle teens  (n = 7) | Late teens  (n = 7) | Total  (n = 21) |
| --- | --- | --- | --- | --- |
| ***1. Disclosure preferences*** |  |  |  |  |
| *1.1 Early vs. late disclosure* |  |  |  |  |
| Early | 5 | 4 | 7 | 16 |
| Late | 0 | 1 | 0 | 1 |
| Before hormonal therapy | 7 | 7 | 7 | 21 |
| ***Advantages of early disclosure*** |  |  |  |  |
| Better compliance with early treatment | 1 | 1 | 1 | 3 |
| Gradual adjustment to living with KS | 0 | 2 | 3 | 5 |
| ***Disadvantages of early disclosure*** |  |  |  |  |
| Being bullied by peers | 0 | 1 | 0 | 1 |
| Earlier feeling of disappointment, anxiety, stress, inferiority complex | 1 | 1 | 1 | 3 |
| ***Advantages of late disclosure*** |  |  |  |  |
| Bypassing years of being bullied by peers | 1 | 0 | 0 | 1 |
| Bypassing years of sadness/disappointment | 2 | 0 | 0 | 2 |
| ***Disadvantages of late disclosure*** |  |  |  |  |
| Delayed treatment, effect on secondary sex characteristic development | 4 | 0 | 0 | 4 |
| Shock leading to possible suicide attempt | 1 | 0 | 1 | 2 |
| Left in uncertainly of what is wrong with their body (delayed secondary sex characteristics) | 0 | 0 | 3 | 3 |
| Much more difficult to accept the diagnosis | 0 | 0 | 5 | 5 |
| *1.2 Total vs. partial disclosure* |  |  |  |  |
| Total disclosure | 3 | 4 | 3 | 10 |
| Partial disclosure | 4 | 3 | 4 | 11 |
| ***2. How would they like the disclosing persons to express themselves*** |  |  |  |  |
| Show empathy, cheer up, give comfort | 4 | 4 | 2 | 10 |
| Neutral and relaxed manner | 1 | 3 | 5 | 9 |
| ***3. Words to avoid using*** |  |  |  |  |
| Infertility | 0 | 1 | 0 | 1 |
| Abnormal, dangerous, incurable | 2 | 0 | 2 | 4 |

KS: Klinefelter syndrome.

**Supplementary Table 3.** Unaffected adolescent males’ viewpoints on the EFFECTS of disclosing Klinefelter syndrome to affected children

| Open-ended questions | Early teens  (n = 7) | Middle teens  (n = 7) | Late teens  (n = 7) | Total  (n = 21) |
| --- | --- | --- | --- | --- |
| ***1. Feelings when hearing “extra female chromosome”*** |  |  |  |  |
| Disappointed, shocked, discouraged, sad | 4 | 0 | 0 | 4 |
| Anxious, worried, confused | 0 | 4 | 0 | 4 |
| Neutral | 0 | 2 | 0 | 2 |
| This word should not to be mentioned | 0 | 0 | 7 | 7 |
| Afraid of gender deviation | 0 | 1 | 0 | 1 |
| ***2. How does knowing that they have extra female chromosome affect them?*** |  |  |  |  |
| Afraid of being bullied | 2 | 2 | 2 | 6 |
| Afraid of being discriminated against by peers | 2 | 2 | 0 | 4 |
| Scared to do things in daily life | 1 | 0 | 1 | 2 |
| Neutral/not afraid of getting bullied | 0 | 2 | 1 | 3 |
| Effect on school performance (no) | 7 | 7 | 7 | 21 |
| ***3. Does having KS affect self-confidence?*** |  |  |  |  |
| Yes | 4 | 4 | 7 | 15 |
| No | 3 | 3 | 0 | 6 |
| ***4. How does having a small penis affect their future plans of having girlfriend and marriage?*** |  |  |  |  |
| Will have a girlfriend | 5 | 6 | 7 | 18 |
| Will not wish to have a girlfriend | 0 | 1 | 0 | 1 |
| Will get married | 0 | 0 | 5 | 5 |
| Do not wish to get married | 0 | 0 | 2 | 2 |
| ***5. How does the word “infertile” affect their feelings?*** |  |  |  |  |
| Sad, shocked, hurtful | 4 | 3 | 0 | 8 |
| Neutral | 0 | 4 | 0 | 4 |
| The word “infertile” should be avoided | 0 | 0 | 7 | 7 |
| ***6. How does being infertile affect their future plans of having girlfriend and marriage?*** |  |  |  |  |
| Will have a girlfriend | 0 | 6 | 7 | 13 |
| Will not wish to have a girlfriend | 7 | 1 | 0 | 8 |
| Will get married | 0 | 6 | 5 | 11 |
| Do not wish to get married | 7 | 1 | 2 | 10 |
| Inform partner before proposing | 0 | 6 | 5 | 11 |
| ***7. How would they cope with the unexpected information of having a KS diagnosis?*** |  |  |  |  |
| Telling oneself that the condition is treatable | 4 | 1 | 1 | 7 |
| Comforting themselves | 1 | 4 | 4 | 9 |
| Consult a psychiatrist as needed | 0 | 1 | 0 | 1 |
| ***8. Keep the KS diagnosis secret or not?*** |  |  |  |  |
| Tell their parents/relatives | 7 | 6 | 7 | 20 |
| Share with their close friends | 0 | 0 | 7 | 7 |
| Share with their girlfriend | 0 | 4 | 7 | 11 |
| ***9. How would they react to a friend with KS?*** |  |  |  |  |
| Comfort the individual, empathy | 3 | 5 | 7 | 15 |
| With normality, as usual | 4 | 7 | 0 | 11 |

KS: Klinefelter syndrome.

**Supplementary Table 4.** Parental viewpoints on the disclosure of Klinefelter syndrome to their child

| 1. When to disclose |
| --- |
| *Early:* Advantages: Gradual adjustment to the diagnosis; avoiding shock (4) |
| Disadvantages: The child may ask questions that are difficult to explain clearly (1); the child may feel unfortunate in having a KS diagnosis (1) |
| *Late:* Advantages: mature enough to understand information |
| Disadvantages: Feeling bad/disappointed that the parents has hidden the truth; |
| loss of trust in their parent (2); difficult accepting the unexpected diagnosis (1) |
| 2. How the parent planned to tell their children at the first time of disclosure |
| Total disclosure (7); partial disclosure (3) |
| 3. What information the parent planned to tell their children |
| Name of the condition 47,XXY and how it can affect the child, focusing on physical health;  external appearance if left untreated; available treatment and good outcome; infertility issue |
| 4. Easiest vs. most difficult topics to explain to the child |
| Easiest: effect of KS on physical health/appearance (1); infertility (4) |
| Most difficult: effect of KS on physical health/appearance (4); infertility (6) |
| 5. Predicted response/reaction of the child after being disclosed |
| Accept it (4); unpredictable (3); worried (4) |
| 6. The most important factor to help their child in coping with the diagnosis after being told |
| Parent-child good/close relationship (6); reassurance that KS is not serious/fatal condition; |
| remind the child that he had lived normal life with KS and it could remain so (2) |
| 7. Red flag signs indicating a call for help |
| Absence from school; depression; avoidance of social activity |
| 8. Help that parents needs from doctors to support their disclosure |
| Treatment to ensure normal male physical appearance and prevention of enlarged breasts in their child (10) |
| Help to explain the necessity of hormonal therapy (10) |
| Counseling aids such as videos or brochures suitable for children (5) |
| 9. How did the information from the adults with KS and unaffected adolescents affect their plans for disclosure? |
| Felt less anxious and had more confidence to disclose the diagnosis to their child (7) |
| Changed their mind from late to early disclosure (7) |
| Learned skills and wording to use and avoid; parents prefer “47,XXY” to “KS” (10) |

KS: Klinefelter syndrome.
